# Supplementary material for: Economic intimate partner violence and its co-occurrence with physical and/or sexual intimate partner violence among youth: Mixed-methods findings from Nairobi, Kenya
Source: PLOS Glob Public Health. 2026 Apr 28;6(4):e0006351. doi: 10.1371/journal.pgph.0006351 (PMC13123958; doi:10.1371/journal.pgph.0006351)
Supplement: Attachment — Submitted filename: PGPH-D-25-02808-comments.docx [file pgph.0006351.s002.docx]

Economic IPV and co-occurrence with physical and/or sexual IPV among youth

1. Introduction: I would have liked some discussion around:
   a. how and why you think economic IPV intersects with sexual and physical IPV
   b. Differences around household economic status and economic IPV
   c. I think we should also talk about gender as well as gender norms as umbrella structures which influence all forms of IPV and bring in the SES as an intersectional variable.
   d. Why do you think SEA is the best fit for assessing economic IPV and the overlap with physical and sexual IPV?
2. Methods:
   a. Did you collect this data among young men as well or was it only young women?
   b. You say you measure economic IPV through a subset of 4 items, but then say you used a 14-item partner economic abuse scale but in the introduction you say you were going to validate the SEA (as per the introduction) which is a 28 item scale?
      i. Economic IPV was measured via a subset of four items selected from the SEA2, a 14-item partner economic abuse scale previously validated in the US and Iran – This sentence is confusing.
   c. It would be nice to see what the qualitative interviews covered, what questions were asked and what was analyzed?
3. Results:
   a. I wouldn’t show the bivariate associations
   b. Your results are structured very nicely!
4. Discussion
   a. I would I have likely more discussion about how these results compare to literature – what is comparable what is not. Why you are seeing these associations?
   b. It would be nice to also have a conclusion.
