# Supplementary material for: Economic intimate partner violence and its co-occurrence with physical and/or sexual intimate partner violence among youth: Mixed-methods findings from Nairobi, Kenya
Source: PLOS Glob Public Health. 2026 Apr 28;6(4):e0006351. doi: 10.1371/journal.pgph.0006351 (PMC13123958; doi:10.1371/journal.pgph.0006351)
Supplement: Attachment — Submitted filename: Replies to reviewers.docx [file pgph.0006351.s003.docx]

Dear *PLOS Global Public Health* Editor and Reviewers,

Many thanks for your continued review of our manuscript "*Economic intimate partner violence and its co-occurrence with physical and/or sexual intimate partner violence among youth: Mixed-methods findings from Nairobi, Kenya.*” We found the reviewer inputs most helpful and are pleased to provide a revised manuscript that we feel has been improved through this process. Please see point-by-point responses below and tracked within the manuscript. Thank you again for the thoughtful feedback.

**Reviewer #1:**

1. **Introduction: I would have liked some discussion around:
   a. how and why you think economic IPV intersects with sexual and physical IPV**

Thank you - we have expanded on this in the first paragraph. [199-204, tracked version]

**b. Differences around household economic status and economic IPV**

We have clarified this in the last sentence of the first paragraph. [151-153]

**c. I think we should also talk about gender as well as gender norms as umbrella**

**structures which influence all forms of IPV and bring in the SES as an intersectional variable.**

We have added an entry statement on this before going into IPV. [140-142]

**d. Why do you think SEA is the best fit for assessing economic IPV and the overlap with physical and sexual IPV?**

We have added to the intro. [170-171]

2. **Methods:
   a. Did you collect this data among young men as well or was it only young women?**

Thank you for highlighting that this needed clarification – we have edited the methods. [226-229]

**b. You say you measure economic IPV through a subset of 4 items, but then say you used a 14-item partner economic abuse scale but in the introduction you say you were going to validate the SEA (as per the introduction) which is a 28 item scale?**

Thank you for pointing out the confusion here. We have revised the methods to be clear that we did not test/validate the full SEA2 scale, but rather pulled 4 items from the scale to apply a shortened, revised version. [252] This is a limitation of our study- and we have also added this into the limitations section of the discussion. [739-743]

**i. Economic IPV was measured via a subset of four items selected from the SEA2, a 14-item partner economic abuse scale previously validated in the US and Iran – This sentence is confusing.**

We have edited to enhance clarity. [252]

**c. It would be nice to see what the qualitative interviews covered, what questions were asked and what was analyzed?**

We have added in additional information on the IDI data collection and qual data analysis to the methods section. [243-246]

3. **Results:
   a. I wouldn’t show the bivariate associations**

Thank you for this suggestion. We understand the sentiment and that it adds tables, but we respectfully ask to keep this in to make our paper more accessible for readers with less quantitative experience.

**b. Your results are structured very nicely!**

Thank you!

4. **Discussion
   a. I would I have likely more discussion about how these results compare to literature – what is comparable what is not. Why you are seeing these associations?**

We have added in comparisons/ more citations throughout the discussion.

**b. It would be nice to also have a conclusion.**

Thank you – we have adjusted our last paragraph, so it is more conclusive. [786-791]

**Reviewer #2:**

**This manuscript measured economic intimate partner violence (IPV) and its co-occurrence with physical and/or sexual intimate partner violence among young women in Nairobi, Kenya. The manuscript is ambitious in its objectives and focussed on economic IPV is an understudied (compared with other forms of IPV). The study applied a mixed methods approach with a strong quantitative part, noting some disconnect of the qualitative evidence in explaining quantitative results. The comments seek to overcome this limitation and strengthen the manuscript further.

Major comments:
Background
• (115-146) The third paragraph encompasses different themes – moving between methods and evidence. Proposing to improve the flow and structure of the narrative beginning with the definition and measurement of economic IPV; tools and adaptations of the SEA in Africa; the prevalence of different forms of IPV in Africa and Kenya; and then highlighting the vulnerability of young women in this context and evidence gap(s)**

Thank you for this comment. We have restructured slightly, but felt it important to report the prevalence findings alongside the measures used, as they should be not comparative and to highlight the importance of measurement for this understudied topic. [176-182, tracked version]

**Methods:
• (166-175) Please report only the number of female participants (Rounds 1 and 4) since male data was not analysed. Clarify who conducted the interviews, where, how long?**

We have updated the sample numbers to reflect the young women only. We also added information on the survey administration. [228-234]

**• (201-207) Please clarify the number of items used for assessing “Past-year sexual and/or physical IPV”. It appears as if only one question was used for each physical and sexual IPV respectively, which would not be best practice.**

We have clarified that we only had two items (one physical, one sexual) within the survey to construct the contact IPV measure. [272]

**• (242-243) Please explain reasons for two different multivariable multinomial regression models and whether the full sample or only sub-sample of those with any IPV experience were included in the model with ‘economic IPV only’ as the reference group (as ‘no IPV’ estimates are not reported).**

Thank you for flagging where more clarification was needed. We added clarification that both models were run among all women, and wrote out the comparisons that each model explores. Since “economic IPV only” vs. “no IPV” is tested in both models, we only present it once in the table. We hope these additions to the methods section increase clarity for the reader. [316-319]

**• (254-261) “Qualitative analysis” appears to be part of “Statistical analysis”, please change level of this sub-heading. Please explain what qualitative data analysis approach used to analyse qualitative data and provide more details about analysis process after coding.**

We have updated the header and edited our text to clarify analysis process. [338]

**Results - quantitative:
• Consider starting Results with the description of the sample (currently 283-289).**

We have made this adjustment. [372-378]

**• Kindly explain the results of the “exploratory factor analysis of the four economic IPV items” in accessible (non-statistical) language (273-274).**

We have edited to increase accessibility. [381]

**Results – qualitative:
• Focus: The qualitative data has a strong focus on gender power relations whilst the survey did not assess gender norms and power but demographic and economic agency measures as covariates. To avoid the disconnect, the narrative part may provide illustrative examples of young women’s limited economic agency (e.g., reworking Theme 1), economic IPV (e.g., reworking Theme 2), and the overlap between contact and economic IPV (currently missing); explore why some women experience economic IPV without any contact IPV (currently missing); and illustrate the identified IPV risk factors (currently missing).**

Thank you for this important point. The overall project for which this analysis draws is focused on broader wellbeing, violence, reproductive health, and gendered behaviors, rather than economic abuse specifically. The qualitative data collection was not specific to economic abuse or the overlap of economic abuse with other forms of contact IPV – rather, the IDIs were quite broad covering topics of relationships, women’s status, violence, help-seeking, and gendered behaviors/ decision making. Within these discussions with women, we identified themes that we found relevant in terms of providing context to the normative environment in which economic abuse operates. We have added clarification on this to the methods regarding data collection and analysis for the qualitative component, and hope this helps clarify the role of the qualitative section for this paper. [243-246, 344-347]

We appreciate the suggestion to reframe the themes so that they better reflect the quantitative data, and have done so in in the updated version. We had previously taken out the section on economic IPV and contact IPV overlap since it was so sparse in the qualitative data, but have re-entered it here, being clear that this was only reflected within a few participant’s experiences. [477-658]

**• Good to see quotes used to back up the narrative provided. However, the labels are not unique, making it difficult to assess if a few participants were quoted repeatedly (e.g., Female IDI participant, 17 years old, IPV data not available) or if quotes were taken from across the sample. Consider adding participant ID (1-15). See also below.**

We have added unique identifier ids throughout; 6 women are represented across the quotes.

**• Noting an overlap between Themes 1 and 2 and 3 around financial decision making. Themes may build on each other but should be independent.**

We hope the new framing and added theme addresses this.

**• Disclosure of economic IPV: Theme 2 provides examples of economic IPV experienced by young women. However, none of the respondents had reported economic IPV in the survey. For example, a participant (375-379) reported economic IPV (item 1+4) whilst the label suggests that she reported only contact IPV in the survey, undermining the validity of the shortened SEA and results.**

Thank you for this important point. While these women replying “never” to the quantitative questions on economic IPV may be a comment on the validity of the quantitative assessment, it could also speak to specificity of the measure by partner. The quantitative measure askes only about the most recent/main relationship, while the qualitative discussion is more open to other partnerships. The specific participant mentioned is unmarried, and we’ve found from our survey that multiple partnerships among youth are very common; over a quarter of sexually active youth in Nairobi have more than one sexual partner. It could be that she has not experienced economic IPV in her main partnership, but is describing an experience with a different partner in which she experienced economic IPV. We have added a footnote.

**• Money equals decision making power: Quotes (411-420) are limited in supporting the claim that financial independence provides women with the means to escape 409 abusive relationships (408-409). Instead, these quotes seem to highlight that power to make decisions rests with people who have money – most often men. However, this does not come out clearly in the narrative.**

Thank you- we have added this point. [637-638]

**Discussion
• Mainly summary of key findings. Please discuss, compare with existing evidence.**
Thank you, we adjusted the full discussion section so there are more interpretation and comparisons to existing evidence.

**Minor comments
Abstract:
• Aim: Consider reducing length by reporting the research aim instead of the objectives
• Methods: More detail needed, e.g., data collection
• Results: Consider adding a result for Objective 3
• Recommendations: Repeats Results, could be shorter**

Thank you; we have updated the abstract to read more concisely. [58-80]

**Background:
• (106-107) “Partner economic abuse has been identified as a form of IPV within the past decade yet remains understudied” needs a Reference.**

We have added an accompanying reference. [149]

**Methods:
• (177-178) The specific date (01/07/2023-15/07/2023) may not be needed, consider replacing with “in July 2023”.**

We clarify that the dates are included at the request of the journal.

**• (183-184) “Purposive sampling was used to ensure representation across gender, age, and violence experience.” Duplication (178-179), consider removing.**

Thank you; we have deleted this duplication.

**• (188-189) “a 14-item partner economic abuse scale previously validated in the US and Iran (12, 13)” Duplication (128-129), consider removing.**

Thank you; we have deleted.

**• (194) “mPesa” please add “(mobile money)” and (196) improve the definition for those unfamiliar with mobile money systems.**

We clarify inclusion of a line stating “meaning mobile money” [271]

**• (209) Consider a better term, maybe Overlap of IPV.**

We have added. [285]

**• (213-220) Consider separating “demographic variables” from “economic agency measures”.**

We have grouped as demographic vs. economic agency under “independent variables.”

**• (225-226) Please explain and reference the threshold for inter-item correlation to be above 0.3.**

We have added this reference. [304]

**• (268) Please report a range of USD as the exchange rate fluctuated a lot during this period.**

We have reported this range. [358]

**• (269) Please justify why informed consent from minors without consent from guardians was considered ethical/sufficient.**

Thank you for this important point; we added in the language from our IRB, citing Kenya Government policy guidance on consent for youth ages 15-17. [365-369]

**Results - quantitative:
• Consider better way of reporting the “prevalence across economic IPV” (275-277) starting with any economic IPV followed by “kept from having money you need for necessities (25.5%), having a job (18.2%), and taking out a loan (8.9%), and (4) forced to give money without paying you back (14.6%) (Table 1).”**

We have revised for clarity. [388-397]

**• Also, consider reporting the Economic IPV Scale Mean here, instead of in Methods (198).**

We have adjusted. [385]

**• Consider reporting the overlap of different forms of IPV (299-300) at once after reporting prevalence of either form of IPV (278-279) instead of repeating.**

We have adjusted. [399-401]

**Results – qualitative:
• Some quotes are long and somewhat detached from the narrative. For example, quote (340-345) should follow after “women are left out of discussion on household finances all together” (334); M voice could be removed by adding information to the quote “We don't talk about such things (how money is spend), he handles them…”**

We have adjusted.

**• Quote (347-350) responds to “…showed little interest in questioning it” (337). Noting that this quote shows some level of participation in decision making as partners agreed the man is responsible for providing food (even if the respondent works).**

We have adjusted.

**• Quote (352-354) would follow naturally after “…shaped by observing parents and older generations” (338).**

We have adjusted.

**• Quote (362-367) is not specific to financial decision making (360); it could be shortened to state “I just follow what he says… I can't say my opinion, if he says something, that’s it.”**

We have moved this into a new theme of economic and contact IPV overlap.

**• Quote (381-382) does not add much, consider removing.**

We have removed.

**• Other quotes do not provide enough information, raising more questions. For example (399-402), how far is the job away – does the respondent have to relocate or commute? Despite the show of male power, he could also be concerned about their relationship and/or her safety.**

While this is true, the quote is more focused on his response, rather than expressing his concerns about her taking the job (which may be warranted), he threatens that he is in charge of this decision. We respectively ask to keep the quote/ narrative as is.

**• Quotes (426-438) provide weak support for the relationship between education, employment and financial independence.**

Thank you; we have adjusted and hopefully the quotes are more clear, concise, and linked to the narrative.

**General:
• Consider defining “contact IPV” clearly once at the beginning of the manuscript to reduce repeating introduction of the term (54, 108, 201).**

We have adjusted so that its explanation only comes up in the abstract and introduction.

**• Please check for typos and edit for conciseness and language.**

Thank you for your attention to detail. We have closely edited this resubmission.
