# Supplementary material for: Economic intimate partner violence and its co-occurrence with physical and/or sexual intimate partner violence among youth: Mixed-methods findings from Nairobi, Kenya
Source: PLOS Glob Public Health. 2026 Apr 28;6(4):e0006351. doi: 10.1371/journal.pgph.0006351 (PMC13123958; doi:10.1371/journal.pgph.0006351)
Supplement: Attachment — Submitted filename: Response to Reviewers.docx [file pgph.0006351.s004.docx]

**Dear *PLOS Global Public Health* Editor and Reviewers,**

**Many thanks for your continued review of our manuscript "*Economic intimate partner violence and its co-occurrence with physical and/or sexual intimate partner violence among youth: Mixed-methods findings from Nairobi, Kenya.*” Please see point-by-point responses below and tracked within the manuscript. Thank you again for the thoughtful feedback.**

Journal Requirements:

If the reviewer comments include a recommendation to cite specific previously published works, please review and evaluate these publications to determine whether they are relevant and should be cited. There is no requirement to cite these works unless the editor has indicated otherwise. Please review your reference list to ensure that it is complete and correct. If you have cited papers that have been retracted, please include the rationale for doing so in the manuscript text, or remove these references and replace them with relevant current references. Any changes to the reference list should be mentioned in the rebuttal letter that accompanies your revised manuscript. If you need to cite a retracted article, indicate the article’s retracted status in the References list and also include a citation and full reference for the retraction notice.

**Thank you; we have reviewed and updated our references to ensure they are fully formatted.**

Reviewer #1:

- Abstract says SEA2 on line 58 but doesn’t provide a full-form. It only becomes apparent in the methods (line 65) that this is an IPV scale. Maybe you can bring this description to line 58.

**Thank you; we have fixed this.**

Figure Resubmissions:

While revising your submission, we strongly recommend that you use PLOS’s NAAS tool (<https://ngplosjournals.pagemajik.ai/artanalysis>) to test your figure files. NAAS can convert your figure files to the TIFF file type and meet basic requirements (such as print size, resolution), or provide you with a report on issues that do not meet our requirements and that NAAS cannot fix. After uploading your figures to PLOS’s NAAS tool, NAAS will process the files provided and display the results in the "Uploaded Files" section of the page as the processing is complete. If the uploaded figures meet our requirements (or NAAS is able to fix the files to meet our requirements), the figure will be marked as "fixed" above. If NAAS is unable to fix the files, a red "failed" label will appear above. When NAAS has confirmed that the figure files meet our requirements, please download the file via the download option, and include these NAAS processed figure files when submitting your revised manuscript.

**Thank you; we have re-attached figure 1 that was ran through the NAAS tool.**
